# The economic evaluation of Cystic echinococcosis control strategies focused on zoonotic hosts: A scoping review

**DOI:** 10.1371/journal.pntd.0010568

**Published:** 2022-07-07

**Authors:** Jo Widdicombe, María-Gloria Basáñez, Mahbod Entezami, Daniel Jackson, Edmundo Larrieu, Joaquín M. Prada

**Affiliations:** 1 School of Veterinary Medicine, Faculty of Health and Medical Sciences, University of Surrey, Guildford, United Kingdom; 2 MRC Centre for Global Infectious Disease Analysis and London Centre for Neglected Tropical Disease Research, Department of Infectious Disease Epidemiology, School of Public Health, Imperial College London, London, United Kingdom; 3 Surrey Health Economics Centre, Faculty of Health and Medical Sciences, University of Surrey, Guildford, United Kingdom; 4 Universidad Nacional de Rio Negro, Choele Choel, Rio Negro, Argentina; The First Affiliated Hospital of Xinjiang Medical University, CHINA

## Abstract

**Background:**

Cystic echinococcosis (CE) is a zoonotic neglected tropical disease (zNTD) which imposes considerable financial burden to endemic countries. The 2021–2030 World Health Organization’s roadmap on NTDs has proposed that intensified control be achieved in hyperendemic areas of 17 countries by 2030. Successful interventions for disease control, and the scale-up of programmes applying such interventions, rely on understanding the associated costs and relative return for investment. We conducted a scoping review of existing peer-reviewed literature on economic evaluations of CE control strategies focused on *Echinococcus granulosus* zoonotic hosts.

**Methodology/Principal findings:**

Database searches of Scopus, PubMed, Web of Science, CABI Direct and JSTOR were conducted and comprehensively reviewed in March 2022, using predefined search criteria with no date, field or language restrictions. A total of 100 papers were initially identified and assessed for eligibility against strict inclusion and exclusion criteria, following the Preferred Reporting Items for Systematic reviews and Meta-Analyses extension for Scoping Reviews (PRISMA-ScR) guidelines. Bibliography review of included manuscripts was used to identify additional literature. Full review of the final manuscript selection (n = 9) was performed and cost data for control interventions were extracted.

**Conclusions/Significance:**

There are very little published data pertaining to the cost and cost effectiveness of CE control interventions targeting its zoonotic hosts. Data given for costs are often incomplete, thus we were unable to perform an economic analysis and cost effectiveness study, highlighting a pressing need for this information. There is much scope for future work in this area. More detailed information and disaggregated costings need to be collected and made available. This would increase the accuracy of any cost-effective analyses to be performed and allow for a greater understanding of the opportunity cost of healthcare decisions and resource allocation by stakeholders and policy makers for effective and cost-effective CE control.

## Author summary

Cystic echinococcosis (CE) is a zoonotic neglected tropical disease which predominantly affects poor pastoral communities globally. The parasite cycles between farm dogs and livestock, and is associated with livestock farming and feeding of infected offal to dogs. Although no noticeable clinical signs are seen in livestock, some production losses, such as reduced milk yield and live weight gain may be observed, and offal condemnation at slaughter is common. The disease can also affect people, due to accidental ingestion of parasite eggs on contaminated food and contact with dogs. Human morbidity and mortality occur due to cyst formation in body organs, exerting a substantial health and financial burden to the health sector of affected countries. Control interventions to reduce CE transmission include sheep vaccination and dog deworming. Long-term control programmes are often expensive, and the true costs of such programmes poorly documented. This scoping review aims to examine published literature on the costs of CE control in zoonotic hosts and report detailed costs of individual elements of a control programme, thereby furthering our understanding of the true economic cost of CE control.

## Introduction

Cystic echinococcosis (CE), also known as hydatid disease or hydatidosis, is a complex zoonotic disease, caused in humans by infection with the larval stage of the taeniid cestode species *Echinococcus granulosus* (Batsch, 1786) *sensu lato* (*s*.*l*.). Humans are accidental intermediate hosts, whilst the lifecycle of the parasite comprises canids (definitive hosts, harbouring the adult stages and shedding eggs in faeces) and ungulates (mainly sheep, acting as intermediate hosts). CE is classified as a neglected tropical disease (NTD) by the World Health Organization (WHO) and most commonly affects poor rural communities engaged in pastoral activities. The parasite is globally distributed and found in every continent except Antarctica. In regions of South America, the Mediterranean littoral, Southern and Central parts of the former Soviet Union, parts of Africa, Central Asia, and China, CE is highly endemic and exerts a substantial health and economic impact [1,2]. Livestock owners incur production-based losses both directly from offal condemnation, and indirectly from decreased carcass weight and decreased milk production. In infected humans, the formation of hydatid cysts within body organs can result in morbidity, however clinically diagnosed cases only account for a small proportion of the total number of infected people as CE can remain asymptomatic for years [3]. Mortality also occurs but is less common. Complications arise if a cyst ruptures, predisposing patients to secondary infections, or if cyst development impedes normal physiological processes [4,5]. Direct costs incurred are associated with diagnosis and treatment of the disease (including surgery and hospitalisation costs), and indirect costs accrue through decreases in productivity and loss of work days, although these are poorly documented [6]. Ongoing surveillance and control of CE in both zoonotic (definitive and intermediate) and human hosts, require a considerable investment by affected countries.

CE is one of 20 NTDs which are included in the WHO’s latest (2021–2030) roadmap to end their neglect and attain the United Nations Sustainable Development Goals [7]. The goals proposed for CE in this roadmap are those of achieving intensified control in highly endemic (hyperendemic) areas of 17 countries by 2030 [7]. Although some countries in South America have had considerable success in curtailing CE transmission via implementation of control measures, and resulting reductions in transmission to humans have been documented, there is substantial variability in intervention implementation and outcomes across the region [1]. Despite effective prophylaxis (e.g., sheep vaccination with EG95) and chemotherapy (e.g., dog deworming with praziquantel (PZQ)), without a structured and coordinated control programme, supported both politically and financially by national health authorities, success is unlikely to be significant and sustainable [8].

Elucidating the true burden of NTDs is essential to monitor progress, evaluate the impact of public health interventions, and inform evidence-based policy decisions, as highlighted by the Global Burden of Disease Study [9]. Increasingly, an adjunct to this information is knowledge of the economic impact of a disease and evaluation of the most cost-effective use of resources for the implementation of control interventions aimed to reduce such burden [10]. This is especially important in financially constrained settings, where prudent and targeted allocation of resources has the potential to exert the greatest impact. Although several studies have provided a comprehensive assessment of production losses associated with CE in various geographical locations [6,11–15], there is little published literature on the cost of control programmes and interventions against CE.

Here, a scoping review of the existing literature is conducted on the economic evaluation of control strategies for CE with the aim to present evidence on the costs and consequences (in terms of disease reduction) of previously utilised control strategies. Specifically, the aims are to: (1) identify and examine the selected studies against a set of good-practice guidelines for economic evaluation and cost effectiveness; (2) extract cost data for control interventions of CE in zoonotic (definitive and intermediate) hosts; (3) summarise and assess key knowledge and identify gaps in research.

## Materials and methods

A scoping review was conducted to identify studies that evaluated the cost of control programmes and interventions in zoonotic reservoirs against CE. The methods for this review follow the Preferred Reporting Items for Systematic reviews and Meta-Analyses extension for Scoping Reviews (PRISMA-ScR) guidelines.

### Literature search and study selection

Online database searches were conducted in PubMed, CABI (Centre for Agriculture and Bioscience International) Direct, Scopus, Web of Science and JSTOR (Journal Storage) during March 2022. The following search terms were used ((echinococc*)|(hydatid) AND (economic evaluation)) AND (cost).

The truncated search term echinococc* was used to capture articles containing the term echinococcosis or echinococcus, and the Boolean operators “OR” and “AND” were used to combine sub-headings and search terms. The key word “cost” was used to capture all articles relating to cost effective, cost effectiveness, cost benefit, cost reduction, and cost analysis. All database searches were identical, with no date, field or language restrictions applied to the search. Duplicate articles were removed, and an initial screening of returned search results based upon title and abstract was conducted. Articles were excluded if they did not pertain to CE caused by *Echinococcus granulosus*; did not relate to animal health; or had no full text available. A full manuscript review was conducted on the remaining articles. Studies were chosen for this review providing they met the inclusion criteria of explicitly provided costings for CE control or interventions. Human health metrics or control interventions focused on humans were not considered (except data pertaining to education programmes given as a sequela to zoonotic host interventions, which were extracted alongside other costings data). Papers only providing economic losses were excluded as the aim of this review was to try and establish the cost and benefit of control interventions against CE in zoonotic reservoirs, and to evaluate intervention strategies for financial viability and sustainable control.

A subsequent search of the bibliographies of the articles selected for full review was also conducted, using the same inclusion and exclusion criteria applied to the original search. The search and subsequent analysis were carried out by the primary author of this review.

### Cost extraction from the literature

Cost data are initially presented in their original format, as per the currency and year of publication. In subsequent analyses, values are converted from their original currency to USD using exchange rates given for the year the cost was incurred. Adjustment for inflation to the year 2020 was applied to the unit cost for all studies (the publication year of the most recently published study) after currency conversion, if required. Where the date was not specifically documented or the study period not given, the year of publication was used as the base year for inflation. Inflation rates were calculated using the R package “Price R” [16].

## Results

A total of 100 studies (PubMed n = 42, CABI Direct n = 17, Scopus n = 18, Web of science n = 9, JSTOR n = 14) were identified in the initial database search. The titles and abstracts of all the identified papers were examined for relevance and inclusion criteria. 27 duplicate studies were removed. Of the remaining 73 manuscripts, 42 were excluded because they were not specific to *E*. *granulosus*, no animal health information was given, e.g., only human health data were provided, or no control interventions were described. A full manuscript review was carried out on the remaining 17 papers. Three studies were excluded as no full text was available. A further six studies were excluded from analysis as no explicit control interventions costings were given, leaving eight studies which fulfilled the inclusion criteria.

**Fig 1:**
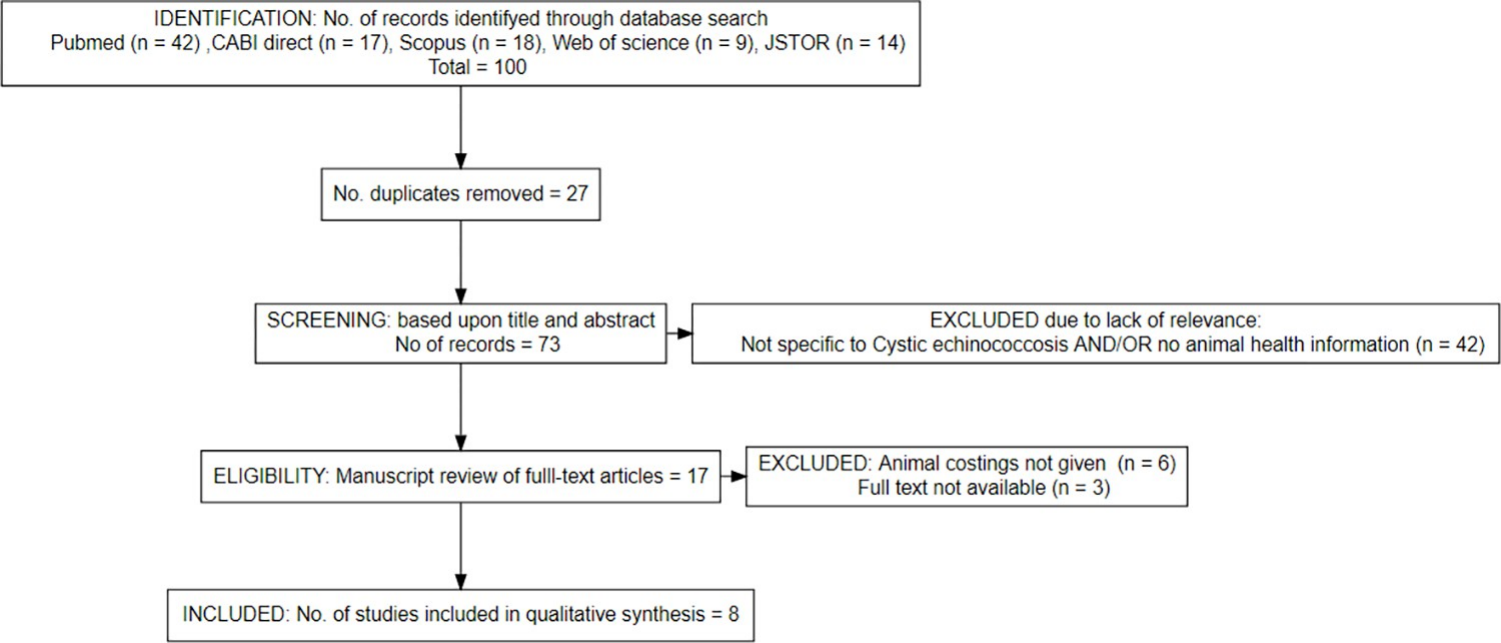
PRISMA flow chart of manuscript selection process for cost of control programmes and interventions against Cystic Echinococcosis in zoonotic reservoirs (definitive and natural intermediate hosts). A total of 100 studies were identified in the initial database search. Duplicates were removed and studies were assessed against the inclusion criteria, resulting in 17 articles being eligible for full manuscript review (six were excluded as no costings for animal health control were given). Eight manuscripts fulfilled the inclusion criteria and were selected for further analysis.

The selection strategy for manuscripts following PRISMA guidelines is shown in Fig 1.

The bibliographies of papers suitable for inclusion were scanned for studies which fulfilled the original inclusion criteria and were not originally retrieved via database search. A further 34 papers were identified and subjected to the same inclusion/exclusion process as previously described, of which nine papers were excluded as they were duplicates, four papers were not able to be found in the databases or via an online search engine, and eight studies were removed as no full text was available. Full manuscript review excluded a further eight papers for lack of relevance, and four due to no explicit control programme costs given. The remaining paper, which satisfied the inclusion criteria, was shortlisted alongside the preceding eight papers, for further analysis.

### Summary characteristics of selected components of the economic evaluation of control for Cystic echinococcosis

With the predefined inclusion and exclusion criteria outlined in this review, Table 1 presents a comparison of summary characteristics from the final selection of articles (n = 9), including study setting, diseases included, animal species targeted for control, and whether production losses were documented.

**Table 1:** Summary characteristics of economic evaluation studies for the control of Cystic echinococcosis.

STUDY NUMBER	LEAD AUTHOR [Reference]	STUDY SETTING	DISEASES STUDIED	TARGET SPECIES FOR CONTROL	PRODUCTION LOSSES GIVEN
(1)	Attanasio and Palmas 1984 [17]	Sardinia	Cystic echinococcosis	Sheep, dogs	Sheep milk production losses
(2)	E. Larrieu et al. 2000 [18]	Argentina	Cystic echinococcosis	Dogs	None
(3)	Jimenez et al. 2002 [19]	Spain	Cystic echinococcosis	Sheep, dogs	None
(4)	Battelli 2004 [20]	Multi*	Cystic echinococcosis	Dogs	None
(5)	Budke et al. 2005 [21]	Shiqu County, Sichuan, People’s Republic of China	Alveolar echinococcosis Cystic echinococcosis	Sheep, goat, dogs	Liver condemnation Carcass weights Production losses Fleece reduction
(6)	Battelli 2009 [22]	Multi*	Cystic echinococcosis	Sheep, goats, dogs	Offal condemnation Production losses Wool losses Carcass weights
(7)	Zhang et al. 2009 [23]	China	Cystic echinococcosis	Dogs	None
(8)	El Berbri et al. 2020 [24]	Morocco	Rabies Visceral leishmaniasis Cystic echinococcosis	Dogs	None
(9)	Cassini et al. 2021 [25]	Italy	Cystic echinococcosis	Dogs	Offal condemnation & reduced offal weights Sheep reduced weight gain Cow reduced milk production Carcass weights

*review discussing multiple control programmes

Causative agents of CE and AE respectively—*Echinococcus granulosus*, *Echinococcus multilocularis*

Summary characteristics of control interventions and health outcomes from the articles are presented in Table 2. Programme types are defined as ‘eradication’, whereby the aim is to reduce CE disease prevalence to zero, ‘control programme’, defined as an established and on going programme aiming to reduce CE disease prevalence with an unspecified duration, and pilot studies, whereby an intervention strategy is being trialed over a fixed time period (usually <5 years). Control interventions used, health outcomes quantified, and the perspective from which the economic evaluation was conducted are also presented. The perspective is the main point of view considered when deciding which costs and benefits are to be included in an economic analysis. Perspectives specific to animal health programmes are sparce, therefore the ‘programme perspective’ [26], whereupon outcomes and costs experienced within that programme alone are considered, along with the widely accepted human health perspectives: societal, health and social services, a specific healthcare provider, and patients and their families. Information on whether further analyses were presented, discounts used, or sensitivity analysis performed, are also extracted and presented in Table 2.

**Table 2:** Summary characteristics of control interventions and health outcomes for the control of Cystic echinococcosis.

STUDY NUMBER	TYPE OF PROGRAMME	CONTROL INTERVENTION COMPARED	HEALTH OUTCOME	PERSPECTIVE	ANALYSIS	DISCOUNT	SENSITIVITY ANALYSIS
(1)	Eradication	Do nothing Anthelmintic prophylaxis owned dogs Stray dog population culling	Potential years of life lost and gained Reduced disease infestation	Societal	None	Yes	No
(2)	Control programme	Anthelmintic prophylaxis owned dogs Surveillance with arecoline*	Canine prevalence Disease prevalence—echo survey	Societal	CBA	No	No
(3)	Control programme	Anthelmintic prophylaxis owned dogs Sanitary pits for offal and culled sheep disposal Stray dog population culling PM surveys of stray dogs culled	Canine prevalence Ovine prevalence Human prevalence	Societal	Health costs saved	No	No
(4)	Control programme	Numerous [review of published work]	Canine prevalence Live weight gain—ovine Years of life gained	Societal	None	No	No
(5)	Control programme	Anthelmintic prophylaxis owned dogs Baiting stray dogs [PZQ] Sheep vac programme** Goat vac programme**	Cost per DALY averted Reduced disease incidence	Societal	CBA	No ^ǂ^	Yes–MV
(6)	Control programme	Numerous [review of published work]	Canine prevalence Ovine prevalence Human prevalence Value of milk production gained	Societal	None	No	No
(7)	Pilot study	Dog registration and treatment with PZQ Stray dog population culling Training and education	Canine prevalence Ovine prevalence	Programme	None	No	No
(8)	Pilot study	Dog registration and treatment with PZQ Surveillance with arecoline Health education	Canine prevalence	Societal	None	No	No
(9)	Model^ϴ^	Anthelmintic prophylaxis sheep dogs [Hypothetical scenario appropriate frequency vs real treatment protocol]	Loss of productivity	Societal	Cost estimation	No	Yes

Abbreviation: PM, post mortem examination, Vac, vaccine, PZQ, praziquantel, CBA, cost benefit analysis, MV, multivariate.

Blank cells represent information not deducible

* Arecoline expulsion is used to induce worm expulsion by dogs to ascertain infection presence and worm load.

** EG95 recombinant vaccine against Cystic echinococcosis.

ϴ Integrated epidemiologic and economic model (EEM)

ǂ Discount pertaining to incomes only, none pertaining to animal health costs provided

The publication dates of the studies identified in this review span 37 years, from 1984 to 2021.

They provide costings for the control of CE in countries in Europe (Studies 1,3,9), Africa (Study 8), Asia (Study 5,7) and South America (Study 2). Two of the studies included a review of multiple locations (Studies 4, 6)an eradication programme was described in one study (Study 1), two studies described pilot studies for CE control (Study 7, 8), and five studies detailed control programmes (Studies 2,3, 4,5 6). An integrated epidemiologic and economic model was described in one paper, providing projected estimates for CE control under a hypothetical versus real treatment scenario (9). All studies included costings for the control of CE caused by *E*. *granulosus*. One study (8) evaluated the integrated control of CE alongside two other dog-transmitted zoonoses, namely rabies and visceral leishmaniasis. However, intervention-specific costs for CE were also given, hence the study was included for further analysis in this review. Another study (Study 5) evaluated interventions for CE along with those to tackle the infection caused by *E*. *multilocularis* (alveolar echinococcosis or AE); however, only the CE-specific costings were considered in this review.

The comparison of control interventions varied widely across the studies (Table 2). However, all studies included anthelmintic treatment of owned dogs with PZQ given at varying intervals. Stray dog management consisted of euthanasia in three studies (Studies 1, 3, 7), with only one study including PZQ treatment of stray dogs (Study 5). Education and training were documented as combined control interventions in three studies (Studies 4,7,8). Resource allocation costings for these components were documented in five studies (Studies 1, 2, 4, 7, 8). Sheep and goat vaccination was given as a control intervention in one study (Study 5). Management of sheep offal and culled sheep disposal with sanitary pits was included in one study (Study 3).

Due to the heterogeneity of study objectives and publication year of the studies, a variety of different methods was used to derive programme effectiveness and measure health outcomes. The health outcome most often given was a reduction in canine prevalence (Studies 2–4, 6–8). A reduction in ovine prevalence was also documented (Studies 3, 6,7), as was human prevalence data (Studies 2,3,6). Reduced ‘disease infestation’, potential years of life lost and gained, reduced disease incidence, and years of life gained were also described (Studies 1, 4,5). Production-related health outcomes included value of milk production gained and ovine live weight gain were given in two studies (Studies 2, 6) and estimated in a third using a model-based approach (Study 9).

Further evaluations of cost data given, and methods of those economic analyses performed were sparse in the published articles. A cost-benefit analysis was presented in two studies (Studies 2,5), and health costs saved given in one study (Study 3). Two studies provided a sensitivity analysis by estimating uncertainty through multiple modelling simulations and parameter estimations (Studies 8, 9). In addition, only one study reported discounted costs (Study 1), with the rest of the studies not clearly stating whether discounting or sensitivity analysis had been utilised in their analyses.

Data pertaining to the cost of CE control interventions in zoonotic reservoirs were extracted from each paper and are presented in Tables 3 and 4 in their original currency and year of publication. Where the control intervention has been described in the paper, yet no cost provided, an ‘x’ is marked in the tables. Blank cells represent control interventions which were not described in the paper.

**Table 3:** Data extraction of the costs for the control of CE.

STUDY NUMBER	LEAD AUTHOR [Reference]	STUDY SETTING	SETTING DETAILS	CURRENCY	DEWORMING COST PER DOG	WAGES
(1)	Attanasio and Palmas 1984 [17]	Sardinia	1982	ITL	1328.57	x
(2)	E. Larrieu et al. 2000 [18]	Argentina	1997 Rio Negro	USD	1.7	
(3)	Jimenez et al. 2002 [19]	Spain	La Rioja 1987–2001	USD	x	
(4)	Battelli 2004 [20]	Multi	1997 Rio Negro 1986–1996 Spain	USD PTS	X 62% TPC	X 3.6%TPC (expenses only)
(5)	Budke et al. 2005 [21]	Tibetan Plateau (China)	Shiqu County, Sichuan	USD	0.12	0.12 per dog (vet costs)
(6)	Battelli 2009 [22]	Multi	Sardinia (10 yrs, period not given)	USD	x	x
(7)	Zhang et al. 2009 [23]	China	Hutubi and Wensu, Xinjiang 1987–1994	USD	1.2	1.5 per dog (admin costs staff)
(8)	El Berbri et al. 2020 [24]	Morocco	Sidi Kaeem Province 2013–2014	USD	1.8	x
(9)	Cassini et al. 2021 [25]	Italy	Veneto region 2019	EUR	4	

Abbreviation: Yrs, years, TPC, Total programme cost. Currencies: USD, United States dollars, ITL, Italian Lira, PTS, Spanish Peseta, EUR, Euros.

*Calculated, ’x’ cost mentioned but no value provided in study, blank cells represent no information provided

**Table 4:** Data extraction of costs continued, including total annual costs.

STUDY NUMBER	STAFF ACCOMODATION ^ǂ^	VEHICLE FUEL	OTHER COSTS	EDUCATIONAL MATERIAL ^ǂ^	TOTAL DOG COST PER YEAR	TOTAL COST PER YEAR
(1)	X	X	100M creation of a computerised information system 200M transport + expenses + educational material	X	1328.57* ITL	126.7M*
(2)			Arecoline purgation 7.3 per dog Household distribution of drug	26	37	440,000
(3)						*19,7225
(4)	X	X 1% TPC	Building of kennels 17% TPC + Septic tanks and wells 10% + incinerators 1% = 28%	X 5.7% TPC	37	X 70.7M*
(5)			0.12 per sheep vaccine + 0.12 vet costs 0.24 Bait costs (inc. distribution costs)		0.48	9073 (CI 8044–10,163)
(6)	X	X		X		0.88M*
(7)			Drug delivery 1.5 per dog Surveillance and progress monitoring 0.5 per dog Culling unwanted dogs—no costs given	0.5 per dog	5.2	X
(8)	X	X	Staff + travel costs 24858	8713	13.5	33,371
(9)			Veterinary surveillance costs (per head cattle) Inc. personnel and transport costs 203.54		32	24,000

Abbreviation: Yrs, years, TPC, Total programme cost, ITL, Italian Lira

*Calculated, ’X’ cost mentioned but no value provided in study, blank cells represent no information provided

ǂ provided as a sequalae to animal health interventions

Costs were commonly given in US dollars (USD); however, three papers provided control costs in local currencies (Italian Lira, ITL (Study 1), Euros EUR (Study 9), and Spanish peseta, PTS (Study 6)).

The cost of dog deworming was documented in all the studies. Many other costs pertaining to the control of CE were considered, but were either not provided, or were aggregated alongside other costings. Vaccination cost per sheep and delivery of the vaccine was only given in one study (Study 5). Wages for staff involved in control programme delivery were individually given in four studies (Study 4, 5,7, 9), but were more often aggregated alongside transportation costs or drug administration costs (Study 1, 2, 8).

Total intervention costs per dog and per year were given in six studies (Studies 1, 2, 4, 5–9), or were calculated if the appropriate data were made available in the paper, i.e., deworming tablet cost, number of doses per dog, and number of animals treated per year. The total programme costs per year were given in eight studies (Studies 1–6, 8, 9).

### Description of costs

Given the diversity of socioeconomic development across the study settings described, differing study landscapes, and breath of timespans covered in the included articles, extreme caution should be made when comparing costs. However, to provide a basic overview, some control intervention costs, standardised for inflation and currency, are presented in Table 5. The range of costs given for preventing and treating a dog against CE for a year was USD 0.60–52.15 (mean ± standard deviation = USD 24.09 ± 22.31), with the individual cost of a dog deworming tablet ranging from USD 0.15 to 4.47 (USD 2.40 ± 1.41).

**Table 5:** Costs, adjusting for inflation, for control of CE 2020.

STUDY NUMBER	BASE YEAR	STUDY SETTING	DOG DEWORMING [BASE YEAR]	TOTAL COST/DOG/YEAR [BASE YEAR]	DOG DEWORMING (2020)	TOTAL/COST/DOG/YEAR (2020)
(1)	1982	Sardinia	0.95	0.95	3.03	3.03
(2)	1997	Argentina	1.7	37	2.4	52.15
(3)	2001	Spain	x	x	x	x
(4)	1997	Argentina	x	37	x	52.15
(5)	2005	People’s Republic of China	0.12	0.48	0.15	0.6
(6)	2009	Sardinia	x	x	x	x
(7)	1994	China	1.2	5.2	2.42	10.5
(8)	2014	Morocco	1.8	13.5	1.92	14.4
(9)	2019	Italy	4.48	35.84*	4.47	35.79*

All costs given in USD

* Estimated cost Data pertaining to the number of doses given to each dog per year were not consistently available.

Due to a small sample size with limited quantitative economic data on the costings of CE control available in the published papers, a meta-analysis was not performed in this scoping review.

## Discussion

This scoping review identified nine studies (inclusive of an eradication programme, pilot studies, control programmes, and an economic model) which evaluated the cost of interventions focused on definitive and intermediate zoonotic hosts of CE. The studies were from Europe, Asia, or South America, reflecting the geographical regions where CE is endemic. Although numerous studies discuss the economic burden of CE, published costs for the control and elimination of the disease are lacking. Of the initial 17 manuscripts selected for full review, 6 were excluded as they gave no definitive costings for CE control despite being picked up by the search terms ‘economic’ and/or ‘cost’. This suggests that although the authors identified the importance of an economic evaluation, they were unable to provide it themselves given their data available. The time horizons given in the study analyses were most often for pilot studies and control programme interventions to date (maximum 14 years). Despite being part of the WHO’s latest (2021–2030) roadmap to end their neglect and attain the United Nations Sustainable Development Goals, there is limited data published recently on the economics of CE control, with all except two (Studies 8,9) of the studies published over 10 years ago, suggesting this information may not be made available to stakeholders or quantitatively analysed and subsequently published.

When considering the anticipated costs of CE control in the future, it is important to consider expected cost of alternative control interventions. Only one study (Study 9) provided projections of expected annual costs for CE control in different control intervention scenarios that could be implemented in the future. In that study, estimates for the cost for dog preventative treatments were given under two different scenarios: preventive treatments in place, and expected costs assuming a more appropriate (more frequent) prevention treatment protocol. Projections of expected costs for different control intervention scenarios, such as frequency of dosing (vaccination or worming), and cost per each species targeted for control allow for a greater understanding of the long-term costs of control and the economic viability and sustainability of a longer-term control programme.

Surprisingly, there were no studies identified in this review published from New Zealand or Iceland, countries in which effective control and elimination programmes have been implemented. Iceland and New Zealand were both declared provisionally free of Hydatidosis in 2002 [27], having utilized similar control interventions to those extracted from the study population. Iceland commenced its CE control programme in 1864 with the publication of education material detailing the lifecycle and the role of dogs in transmission of the disease. Subsequently in 1890 a law was passed banning the feeding of sheep offal to dogs. A taxation to dog owners was also implemented at this time, with dog deworming becoming mandatory also. By the early 20^th^ Century, slaughterhouses were built across the country, and it was illegal to kill livestock outside of them [27]. The success of the Icelandic control programme encouraged other island countries to commence their control programmes against CE. In 1938, New Zealand initiated a voluntary education programme, which included advice on feeding dogs, the correct disposal of sheep offal, and arecoline purgation of dogs four times per year, and in 1940, the meat act made offal feeding illegal. In 1959 an independent National Hydatids Council was formed, funded by a dog mandator license fee, paid for by dog owners. In 1972 arecoline screening of dogs was replaced with 6 weekly anthelmintic dog treatment, which was continued for 19 years until the disbanding of the Hydatids Council in 1991. Animal movement control was subsequently implemented by the Ministry of Agriculture in the control programmes ‘consolidation phase’, until 2002 when a declaration of hydatid free status was made in New Zealand. It should be noted that Iceland and New Zealand are both small island nations, and the success of these control/eradication programmes are unlikely to reach the same level of success in other locations given the lack of a maritime border (preventing unofficial animal movements), and geographical and social factors preventing ease of access and implementation of control interventions.

Data for costs are often incomplete, with considerable variation in the type of costs given, and descriptions of how costs were incurred and accrued over time. Most studies were descriptive of an intervention programme protocol, with costs of an intervention given compared to the status quo or ‘do nothing’ scenario as a counter factual. Few studies provided actual costs, and very limited, if any, analyses of cost effectiveness of each intervention.

Cost effectiveness analysis determines the costs and consequences of alternative health interventions, measured as a unit of health change such as disability-adjusted life years (DALYs). Although widely accepted in human health, and utilised by the WHO to measure the global burden of disease [9], the use of DALY’s for zoonotic diseases have been questioned as it fails to capture the complete health and financial burden resulting from production losses in diseased animals [28]. The zoonotic DALY (zDALY) has been used as an alternative health metric, encompassing the monetary value of animals alongside the burden of disease captured by the DALY [29], however its use is currently not widespread, and it was not utilised in any of the studies analysed.

A difficulty when analysing the economic data was that of programme costs being aggregated without a clear breakdown or inclusion policy of what was included in the cost given. An example of this is dog deworming, which was used as a control option in all the studies. Despite being documented, the cost for a worming tablet was not always documented, and details regarding programme delivery and frequency of administration were often unclear. The price of PZQ deworming tablets is relatively inexpensive in the more recent studies, yet there is a wide range of values in the total cost of PZQ per dog, per year. This could be reflective of the expense of individual tablets prior to the expiration of its pharmaceutical patent between 1989 and 1994, after which time, generic formulations became more widely available at a much-reduced price [30]. PZQ was initially produced primarily in Germany (by E. Merck and Bayer Pharmaceuticals), South Korea (Shin Poong Pharmaceuticals), and in China, whereupon it was globally distributed to both the private and public sectors, and by international agencies. Shipping costs and the overall purchase volume will also have contributed to the varying documented costs of the drug, with a country’s purchasing power and ability to buy in bulk (with subsequent bulk discounts) affecting the overall purchase price secured [30]. At the local programme level, the variable costs seen may also be attributable to higher programme delivery costs for more frequent mass drug administration, the details of which are not completely documented in the studies reviewed.

Only one study included cost of sheep vaccination despite its availability since 2006 [31]. This may suggest the presence of very few control programmes utilizing vaccination, or a lack of uptake in some regions due to the cost of the vaccine. Until recently vaccine supplies were donated to some countries by Melbourne University [8] thus costs for this may not have been accounted for.

The overall programme costs documented are affected by the different types of control being implemented and necessary routes of drug administration. Some studies utilised community members for programme delivery, who are unpaid, thus costs such as staff wages and transport were often omitted or considered irrelevant. However, for those utilizing sheep vaccination and sheep deworming, skilled personnel are required, thus the cost of wages must be documented (or estimated) in the total programme delivery costs. Pertinent details of the geography of study areas, such as remoteness and ease of access to the farms was often incomplete too, thus it is prudent not to draw comparable conclusions between different study regions.

To perform a full economic review and analysis, a unit of health outcome needs to be provided as a measurement for the intervention. There was variation in the different health care metrics reported in the studies, some reported an increase in productivity of animal by-products (including milk yields, live weight gain, and reduced liver condemnations), years of life lost and gained, disease incidence, and disease changes seen on ultrasound analyses. More standardised approaches such as disease prevalence are given in the more recently published papers, compared to metrics such as ‘disease infestation’ cited in the oldest of the studies (published in 1982). Reporting of CE in humans and its zoonotic hosts is not universally compulsory in all countries [1]. For NTD’s in general, data collection systems are often not standardized, fragmentary and independently collected by public health, veterinary and stakeholder sectors. This often leads to a lack of reliable qualitative and quantitative data on disease burden [28], with underreporting and gaps in data leading to an underestimation of disease burden in endemic countries. Dog prevalence was the most frequently cited health outcome in the studies analysed; however, diagnostic methods and detailed information regarding the dog population being tested was not always clear.

In several of the CE publications excluded during the manuscript selection process, the control intervention was often being applied to the zoonotic hosts, but the health metric reported pertained to human disease and human disease burden. Given that a reduction in human disease is the intended outcome of a CE control programme, this may be appropriate, but if often leaves some animal health metrics along with their costings unpublished.

With such a variety of health outcomes measured, and only three studies providing an analysis of cost effectiveness in this review, a full economic evaluation and metanalysis was not appropriate to this scoping review. However, the economics of NTD control, alongside a detailed understanding of their epidemiology are pivotal for their sustainable control [32], thus there is great potential for further work in this area as more detailed information becomes available. Required data would include that of the unit cost and number of units used of all items included in a control/pilot programme. This may include drug costs per dose, costs for delivery of the drug including any materials required such as syringes or dosing guns, cost of cold chain storage if required, vehicle and transport costs, and estimated personnel time and wages.

A notable research gap is that of integrated CE control programmes alongside other NTDs. One study considered CE control as part of an integrated control programme with other dog-transmitted zoonoses; however, most of the studies considered CE independently. The combined delivery of multiple health interventions has the potential to maximise intervention coverage and minimise costs [33,34]. Although encouraged for other NTDs such as schistosomiasis, lymphatic filariasis, onchocerciasis, and the soil-transmitted helminthiases, there is limited evidence of those including CE despite its global distribution. Given that NTDs and zNTDs, including CE, predominantly affect impoverished communities with inadequate access to sanitation, healthcare, clean water, and education, the potential for increased cost effectiveness of integrated control programmes could be explored further.

In conclusion, this scoping review has provided a preliminary assessment on the economic evaluation of cystic echinococcosis control strategies focused on zoonotic hosts. There is great scope to widen the knowledge-base of cost effectiveness of different CE control interventions, which is currently lacking in the published literature. To this end, more complete costing data need to be collected and collated. In particular, pertinent details of the costs associated with a mass drug administration programme, such as drug costs, materials for drug administration and number used, cold chain storage, materials and clear study methodologies, and which costs are explicitly included when costs are formulated, would add accuracy to any future cost-effective analysis.

## Supporting information

S1 PRISMA Checklist Preferred Reporting Items for Systematic reviews and Meta-Analyses extension for Scoping Reviews (PRISMA-ScR) Checklist. (DOCX)
